# Physiological Roles of Eumelanin- and Melanogenesis-Associated Diseases: A Look at the Potentialities of Engineered and Microbial Eumelanin in Clinical Practice

**DOI:** 10.3390/bioengineering11080756

**Published:** 2024-07-25

**Authors:** Maria Letizia Terranova

**Affiliations:** Dip.to di Scienze e Tecnologie Chimiche, Università degli Studi di Roma “Tor Vergata”, 00133 Roma, Italy; terranov@uniroma2.it

**Keywords:** bioinspired eumelanin, eumelanin-associated pathologies, microbial eumelanin, supply of engineered eumelanin, eumelanin-based therapeutics, melanogenic microorganisms, radioprotection

## Abstract

This paper aims to highlight the physiological actions exerted by eumelanin present in several organs/tissues of the human body and to rationalise the often conflicting functional roles played by this biopolymer on the basis of its peculiar properties. Besides pigmentary disorders, a growing number of organ injuries and degenerative pathologies are presently ascribed to the modification of physiological eumelanin levels in terms of alterations in its chemical/structural features, and of a partial loss or uneven distribution of the pigment. The present review analyses the more recent research dedicated to the physiological and pathological actions of eumelanin and provides an insight into some melanogenesis-associated diseases of the skin, eye, ear, and brain, including the most significant neurodegenerative disorders. Also described are the potentialities of therapies based on the localised supply of exogeneous EU and the opportunities that EU produced via synthetic biology offers in order to redesign therapeutical and diagnostic applications.

## 1. Introduction

The naturally occurring brown–black pigment eumelanin (henceforth called EU) is the final product of complex biochemical reactions that start from the amino acid L-tyrosine [1].

**Figure 1:**
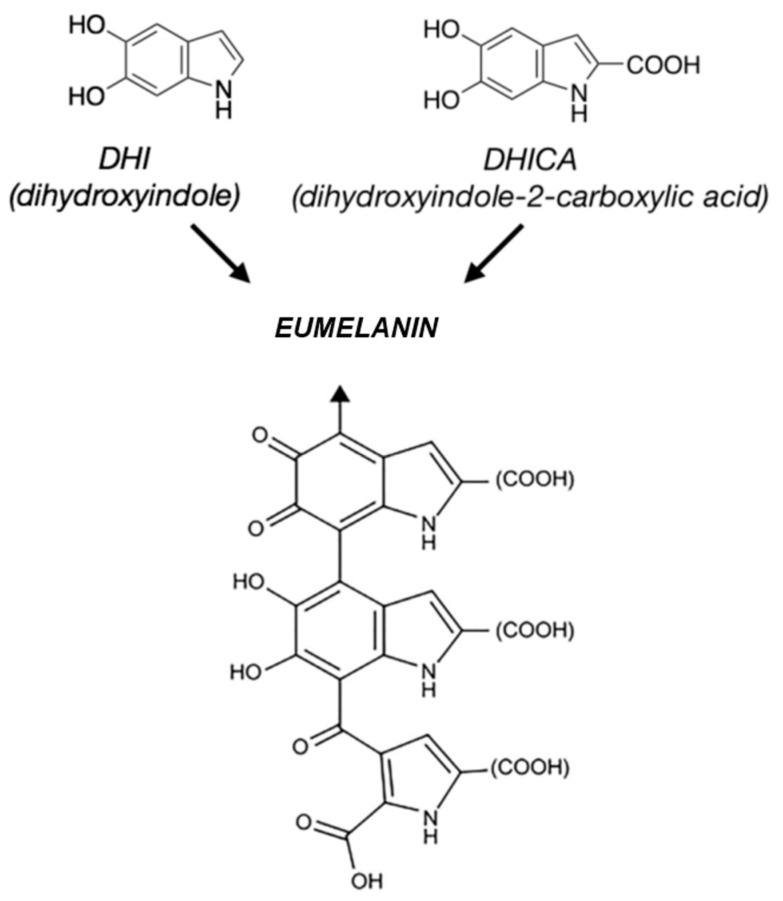
Schematic representation of eumelanin synthesis from DHI and DHICA units [Adapted from Ref [2] (open access)].

This pigment, found in virtually every living organism, is a heteropolymer of indolic nature basically composed of a variable number of 5,6-dihydroxyindole (DHI) and 5,6-dihydroxyindole 2-carboxylic acid (DHICA) units, cross-linked through chemical bonds or physical interactions [2]. Figure 1 shows the synthetic pathway that, starting from its basic building blocks, leads to EU.

**Figure 2:**
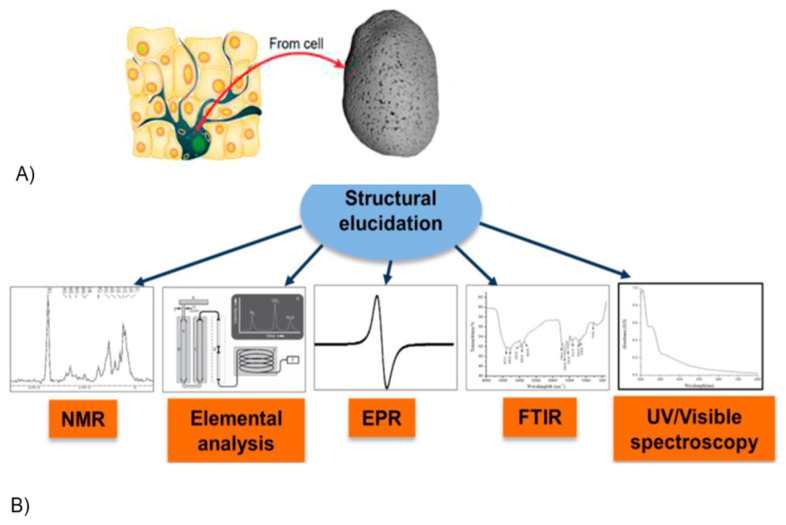
(**A**) Image of a eumelanin granule (right) extracted from a melanocyte (left) [Adapted from Ref. [4], (open access)]. (**B**) Indication of the main techniques used to identify EU and investigate its chemical and structural features [Adapted from Ref. [5] (with permission)].

In mammals, EU enzymatic synthesis occurs through melanogenesis, a complex mechanism carried out by specialised melanocyte cells that generate and deposit pigment granules (Figure 2A) into intracellular organelles known as melanosomes [3,4].

The chemical and structural analysis of EU extracted from animal or vegetal organisms enabled the elucidation of the primary structure of the macromolecule and the development of efficient synthesis strategies. EU can be obtained *in vitro* by several chemical processes, such as the oxidation of tyrosine with H_2_O_2_, or the oxidative polymerisation of 3,4-dihydroxy-L-phenylalanine (L-DOPA) or DHI [2].

The variability in the DHI/DHICA molecular ratios and the stacking of monomers into a variety of sub-structures formed by two, four, five, or six building blocks induce an inherent “geometrical disorder” that, in turn, triggers an “electronic disorder” and accounts for EU’s peculiar functional properties [6,7,8]. 

The many significant studies performed on EU during the past two decades allowed a full spectrum of unique chemical/physical properties to be evidenced. These include its charge transport ability, absorption/conversion of both ionising and non-ionising radiation, its peculiar free radical scavenging activity and related redox behaviour, metal–ion chelation capacity, and ability to stick to metallic surfaces [9]. The discovery of such appealing properties has recently led to EU being considered an attractive material not only for biomedicine, cosmetics, and environmental remediation [10,11], but also for some advanced green technologies such as energetics and bioelectronics [12,13,14,15]. With regard to the field of biomedicine, the challenge is to translate the laboratory results into viable clinical practices. It is to be noted that while EU is opening up new possibilities in various advanced bio-related applications, not all of the physiological activities of EU have been rationally explained and some functionalities remain rather elusive [16].

Though it is well known to non-specialists that EU is the macromolecule responsible for the pigmentation of the mammalian skin, hair, and iris, the remarkable presence of melanocytes in other body parts, including in those not exposed to light, is generally less noticed [17,18,19]. The fact that EU is present in many organs and tissues, including the inner ear, brain, heart, arteries, and ovaries, should not be overlooked, as almost all cells contain precursors of melanin synthesis [20].

This paper aims to highlight the consistent distribution and physiological state of EU in specific locations of the human body in an attempt to rationalise the many and often conflicting physiological roles played by EU on the basis of the peculiar functions performed by this natural polymer. The focus is on the diseases associated with melanogenesis disorders, on the potentialities of therapies based on the supply of exogeneous EU, and on the opportunities that EU produced through synthetic biology offers in the redesign of therapeutic and diagnostic applications.

## 2. Properties and Functions

The biological functionalities of EU are inextricably intertwined with the many outstanding chemical/physical properties that characterise this pigment. The following paragraphs summarise the main properties that drive the physiological actions of this polymer in the skin, eye, ear, and brain.

With regard to optical performance, EU is characterised by a broad absorption spectrum that extends from NIR to UV and by an exponential decrease in the extinction coefficient at higher energies [1,21]. Such features explain the photoprotective action on living tissues exposed to UV rays and the ability of pigmented tissues to absorb and dissipate the energy released by such ionising radiation.

However, while this behaviour has been known for a long time [22], it is only in the last two decades that research has been able to highlight certain aspects of EU’s interaction with UV-Vis radiation and to clarify the mechanism of the decay of optically active states after the absorption of the UV portion of the EM spectrum [23,24,25].

The prevalently non-radiative decay of light-exposed EU occurs through a complex mechanism of thermal relaxation, following two different pathways. The first is a rapid relaxation of the excited states and accounts for the traditional photoprotective function exerted by the pigment [26,27]. The second one proceeds *via* the photogeneration of radicals and triplet states that deactivate on a longer time scale [28]. This delayed relaxation channel makes it possible for the excited macromolecule to be involved in redox reactions that modify the oxidation states of its subunits and eventually trigger pro-oxidant actions. 

EU belongs to the class of semiconducting polymers, but additional remarkable features make it different from the most common ones. 

Besides being a photoconductor [29], this polymer is also a hybrid ionic–electronic conductor [30]. Therefore, EU not only has the peculiar feasibility to increase the conductivity and to swap the direction of electron transfer depending on its redox state, but also the ability to switch its electrical conduction from electronic to protonic and vice versa [31]. Recent studies demonstrated that the relative contribution of these different mechanisms to the macroscopic conductive behaviour of EU depends on its hydration level [30].

EU is also characterised by peculiar paramagnetic properties derived from the unusual presence of two types of radicals, namely, “intrinsic” stationary free radicals centred on carbon atoms, and “extrinsic” free radicals generated by oxidative stresses [29,31,32,33]. The free radical scavenging ability of EU is related to the degree of electronic delocalisation within the polymer backbone [34] and is, therefore, dependent on both the DHICA/DHI ratio and the supramolecular organisation of such building blocks [8]. In particular, the radical scavenging performances of EU rely on the oxidation state of the pigment, which, in its reduced form, quenches oxidative radicals, and in its oxidised form acquires electrons from reductant radicals [35]. Consequently, any alteration of the redox equilibrium between the subunits of this polymer strongly affects its radical scavenging ability, potentially even reversing its antioxidant function [36]. 

The ability of EU to act as an efficient scavenger of radicals and reactive oxygen species (ROS) allows the pigment to counteract oxidative stress caused by any oxidising agent [33] and also accounts for the presence of melanocytes in anatomical parts not exposed to light. 

In this context, it must be stressed that EU is a unique molecule able to offer protection against all kinds of ionising radiation, from X-rays to the α, β, and γ emissions generated by radioactive decay. By means of sophisticated chemical/physical mechanisms, EU is able to absorb and dissipate even highly energetic ionising radiations, quenching the large amount of dangerous free radicals that they produce.

The transduction processes that take place during the interaction of such radiations with EU not only provide effective protection against radiation-generated cytotoxic species [37], but also an unexpected conversion of the energy released by radiation into other advantageous forms of energy. Indeed, it has been highlighted that the EU present in biological systems absorbs and converts energy available for metabolic processes from any type of ionising radiation, including radioactive emissions [38]. 

The increase in the growth activity in melanised organisms exposed to radiation was first noted in melanised fungal communities flourishing in radioactive environments [39]. The activation of this radiosynthesis process, called radiotropism, explains the tendency of the EU-containing cells to strongly attract radioactive species [40,41].

The remarkable ability to bind redox-active metal ions and oxides is a further outstanding ability of EU. The effective metal-chelating activity, an issue relevant in biochemistry, pharmacology, and toxicology, is another effect of the heterogeneous chemistry of this polymer. In effect, EU has the potential to interact with metallic species through the phenolic, aminic, or carboxylic groups of its indole units [42]. These various options give rise to a high variability of binding sites and to marked differences in the affinity for the various metal ions [14]. For some ions, such as Mg(II), Ca(II), and Zn(II), EU acts as an ion exchanger and metal reservoir able to accumulate the metal ions that, under particular conditions, can be also released. In the case of Ca(II), the macromolecule also works as an intracellular buffering system [43]. 

Other metal ions are more strongly bonded to EU and, therefore, remain trapped by melanosomes. This is the case for Fe(II) and Cu(I) ions, which are involved in the mitochondrial electron transport chain [44]. These ions easily undergo reversible oxidation/reduction processes and form complexes with low-molecular-weight ligands. Even if no really “free” Fe and Cu ions exist in the cytoplasm, the complexes that they form are involved in Fenton reactions that catalyse the production of highly reactive hydroxyl radicals from hydrogen peroxide and lipid hydroperoxides [44,45].

By putting into action efficient processes of metal uptake, EU is generally able to contrast the induction of oxidative stress and to protect living cells from cytotoxic effects [43,46,47]. However, a significant build-up of metallic species at specific sites of the macromolecule can modify the concentration of free radicals in the EU fragments and activate dangerous Fenton processes [48].

As has been known for a long time, EU also succeeds in inhibiting the peroxidation of lipid and cardiolipin liposomes induced by Fe(II) ions [49] and by Fe(II)–ascorbic acid systems [50]. 

A deep insight into the biofunctional role played by the interaction of EU with redox-active metals can be found in [44].

## 3. Physiological and Pathological Actions

### 3.1. Skin

The EU present in skin is able to absorb the whole UV–visible portion of the electromagnetic spectrum and to manage UV-generated ROS. Triggered by its unique free radical system, EU puts into action an efficient mechanism of de-excitation [51]. However, the different time-scale of the photochemical and photophysical processes involved in the relaxation of light-excited EU strongly affects its photoprotective function, which can switch to an opposite phototoxic one [22].

As a result, EU exposed to light can perform conflicting functions, and this unexpected behaviour explains why, in some cases, melanised cells are more susceptible to light-induced damage than unmelanised ones [52,53]. These findings allowed the development of an initial understanding of the mechanisms by which UV radiation induces melanomas [24]. 

It is to be noted that, depending on the wavelength, the onset of UV-induced melanomas follows different pathways. In the case of UV-B radiation, cellular damage is produced by the direct excitation of DNA nucleotides and the pigment does not play any role in such a process. Conversely, EU is significantly involved in the cellular damage provoked by UV-A radiation that induces melanomas by means of an indirect chemi-excitation mechanism [54]. The triplet states generated by the UV-A radiation inside the EU backbone de-excite through a non-radiative transfer of UV energy to cellular DNA. In this case, the pigment operates as an energy mediator [55]. This relatively slow oxidative mechanism provides an explanation for previously detected odd effects, namely, the photogeneration of dangerous DNA products and the occurrence of skin pathogenic processes that also occur under dark conditions [56]. 

Overall, it has been highlighted that the decay of the channels activated by UV-Vis light is strongly affected by the heterogeneity of the macromolecule and that occasional modifications of its chemical/structural organisation alter the photophysical and photochemical processes. This alteration can even reverse the protective actions of EU against oxidative stresses, conversely inducing phototoxic effects that can lead to malignant melanomas of the skin and eye [55,57]. It should be noted that melanomas are characterised by an out-of-control growth of melanocytes and a dysfunctional increase in the physiological levels of eumelanin [58].

While the undeniable evidence of a relationship between photoreactivity and melanoma pathogenesis is still under debate, it is now documented that a pigment traditionally thought to be a protector against photo-induced cancers may also play a detrimental role [44].

It must be noted that cutaneous pigments are generated by skin melanocytes. Therefore, any damage occurring in these cells affects EU production and gives rise to different types of pigmentation disorders, from hyperpigmentation to hypopigmentation of a genetic or acquired nature [59]. A congenital disorder of the pigmentary system is albinism, a severe hypopigmentation that can not only affect skin and hair colour, but is often associated with defects of the ocular and auditory systems [60]. In fact, EU is located close to the photoreceptor cells of the eye and ear, where it governs the transmission of functional signals, processing light and sound into sight and hearing [17]. The most common acquired pigmentary disorder is vitiligo, a disease caused by melanocyte loss that afflicts 0.5–1% of the population and is characterised by depigmented skin patches [61]. 

### 3.2. Eye

With regard to the eye, EU is present in both the retinal epithelium and uveal coat [62]. Retinal EU is involved in the photoreceptor physiology and in the protection of ocular tissues against damage produced by exposure to UV radiation, atmospheric oxygen, and environmental pollutants [17]. It has been reported that EU is able to absorb and convert about 99.99% of the UV radiation that hits the eye [61].

Even if the protection against harmful radiation of the EM spectrum is the same function exercised by EU in the skin, there is a remarkable biological difference between eye and skin eumelanin. The latter one is continuously synthesised by epidermal melanocytes, whereas the EU of pigmented eye tissues shows practically no renewal throughout life [63]. Of course, this makes the biological effects of any chemical or structural modification occurring in eye EU as a consequence of injuries or just of ageing more serious [64]. A decrease in ocular pigmentation has a strong impact on the whole visual system, inducing *in primis* photophobia, but also neurological disorders due to the misrouting of the signals sent to the brain and the consequent alteration of visual acuity, eye movement, and stereovision [17].

Age-related dysfunctions of the metal chelation processes are responsible for some eye pathologies that can appear in the elderly [65]. Other effects related to EU disorders, such as the decrease in antioxidant response, contribute to the induction of severe ocular diseases. A reduced ability to quench oxygen-derived free radicals and the consequent peroxidation of phospholipids and of other lipid components of ocular tissues strongly contributes to macular degeneration, cataracts, glaucoma, and diabetic retinopathy [66,67]. 

Retinal EU also plays a role in countering a different types of oxidant agents, namely, radioactive emissions. Here, the radioactivity accumulated in the pigmented tissue, if not exceeding the threshold of irreversible damage, is absorbed and dissipated by a protective molecular mechanism able to enhance DNA repair [68,69]. In this context, an interesting feature of the vision system regards the distribution of administered radiolabelled drugs [70,71]. Due to the EU attraction for ionising radiation and the tendency to strongly bind radioactive sources, in the eye of pigmented animals, radioactive species are found selectively concentrated in the retina [72]. 

### 3.3. Ear

The presence of EU in the hearing organs of mammals varies from species to species. In humans, EU is mainly located in the inner ear, specifically in the cochlea and in the endolymph [73]. Cochlear pigmentation is a determinant in the response of the inner ear to acoustic stimulation. It has been found that darker-skinned individuals and those with brown irises tend to have more inner ear melanin and suffer less noise-induced temporary threshold shift and noise-induced hearing loss than individuals with blue irises or those with albinism. This confirms that cochlear melanocytes play an important role in preserving the hearing physiology under normal conditions and also in exerting a protective role in the inner ear under certain pathological conditions [74]. 

EU is present in various components of the vestibular organs, such as the stria vascularis, dark cells, and the Reissner membrane [67]. The Reissner membrane allows for the selective transport of K(I) and Ca(II) ions, generates the endocochlear potential that drives the key dynamics of potassium-regulating proteins, and is involved in endolymph production. The dark cells pump K-ions into the endolymphatic fluid, whereas the cells of the stria vascular help to maintain the high membrane potential and the K-ion concentration of endolymph [75]. Moreover, due to EU’s ability to act as an ion exchanger and metal reservoir, the vestibular melanised cells regulate the ionic concentrations and the opening of Ca(II) channels, allowing neurotransmission along the nerve fibres that connect the vestibulo-cochlear system to the visual system and induce our perception of balance and equilibrium [76]. 

Hypopigmentation of the inner ear is related to different levels of diseases and is commonly associated with an enhanced susceptibility to noise-produced damage and the reduced spatial localisation of sounds [77]. 

### 3.4. Brain

In the brain of humans, EU is found inside melanocyte organelles, where ≈ 30 nm sized EU granules surrounding a pheomelanin core give rise to so-called “neuromelanin” [78]. This hybrid biopolymer, located mainly in the “substantia nigra”, “locus coeruleus”, and “medulla oblongata”, performs a series of fundamental physiological functions [17]. The outstanding dual ionic/electronic conduction of EU is exploited in neurotransmission [79], with EU being able to scavenge free radicals in counteracting oxidative processes and to sequester redox-active and toxic metal ions in the protection against neurological diseases [80].

However, EU does not always succeed in protecting cells against the oxidative stress mediated by redox-active metals and oxides [81]. The EU protective functions, in fact, can be affected either by an anomalous accumulation of metals, especially Fe, or by the degeneration of melanosomes [48]. Moreover, ageing and other stimuli are found to induce changes in the redox equilibrium between the DHI and DHICA subunits. Due to the strict interconnections among the various EU functionalities, any perturbation of the redox equilibrium results in an alteration of the metal-chelating ability and in the possible occurrence of neuroinflammation and neurodegeneration processes [44,65]. 

In particular, the dysregulation of the mechanisms that control the uptake/release of metal ions is thought to contribute to several neurodegenerative diseases [48]. It has been noted, indeed, that various neurological disorders are characterised by a significative accumulation of Fe ions in specific regions of both the central nervous system and the peripheral nervous system. 

Abnormalities in Fe homeostasis, increased levels of lipid peroxide, and the production of massive amounts of ROS are features found to be associated not only with the most common Alzheimer’s (AD) [82,83,84] and Parkinson’s (PD) diseases [85,86], but also to Huntington’s disease (HD) [87], amyotrophic lateral sclerosis (ALS) [88], and Friedreich’s ataxia (FA), an autosomal recessive hereditary disease [89]. All of these neurological pathologies seem to be related to a nonapoptotic mode of cell death known as “ferroptosis”, a pathophysiological iron-dependent process [90]. 

Modifications of EU chemistry, as well as an uneven distribution of EU in tissues, are found to affect the mechanism of Fe chelation and to unbalance the equilibrium between the uptake and release of the metal [49], activating a cell ferroptosis process [91]. It is to be noted that this mechanism of oxidative cell death is also closely related to other severe non-neurological diseases, such as certain types of cancer, nervous system disorders, ischemia–reperfusion injury, kidney injury, and blood diseases [48].

With regard to Alzheimer’s disease, a further possible role of EU has been suggested, namely, the control of amyloid-beta42 formation and of its aggregation in fibres [92]. 

Table 1 summarises the main diseases of skin, eye, ear, and brain associated with damaged EU or alterations in physiological EU levels. 

## 4. Mechanisms of Action of Exogeneous EU 

A series of studies and research focused on novel therapeutic approaches suggest EU and EU-based materials as reliable tools and multifunctional nanoplatforms for medical diagnostics, regenerative medicine, and nanomedicine [11,99,100,101]. 

The intrinsic photoacoustic properties of EU are exploited to diagnose various diseases by means of non-invasive Photoacoustic Imaging, a modality characterised by higher spatial resolution and sensitivity with respect to other optical techniques [102].

Due to the intrinsic ability to chelate metal ions, melanin and melanin-like materials are finding applications as contrast agents in monomodal imaging techniques, but also in the emerging multimodal methodologies that combine different techniques [102,103].

In regenerative medicine, natural EU and its synthetic analogues are being proposed as promising materials for a series of applications, from the regeneration of bone and cartilage tissues to the repairing of nerve defects. Nanofibrous scaffolds of polyvinyl alcohol incorporating EU nanoparticles have been tested for the myogenic differentiation of skeletal myoblasts [104], whereas nanofibrous composites formed by EU and silk fibroin were found to promote neuronal growth and nerve regeneration [105]. In general, EU-based nanocoatings were demonstrated to increase cell attachment and proliferation on different substrates [106]. 

In nanomedicine, EU-inspired multifunctional nanomaterials are promising nanocarriers for the delivery of therapeutics in various organs and tissues, from kidney [107] to retinal epithelial cells [108]. Moreover, EU-containing drugs can behave like multifunctional platforms able to perform targeted drug delivery under the guidance of imaging techniques and to realise self-monitored anticancer photothermal therapies [109,110].

The ability to respond effectively to a variety of chemical and physical stimuli allows EU to be employed as an exogenous conversion agent for a series of therapies, including photothermal and photodynamic ones [111]. 

It is to be noted that in all of the above-reported applications, EU is being used (or is planned to be used) to replace other materials, aiming to optimise the performance of systems already employed in biomedical fields [112]. However, much more impressive are the approaches that try to replace or implement missing or defective endogenous EU with exogeneous EU. The many aspects of human physiology to which this polymer contributes suggest that such emerging routes would be effective to relieve several diseases related to either innate or stimuli-induced melanocyte degeneration. 

A noticeable number of studies have recently focused on the utilisation of exogeneous EU and its derivatives in new potential therapeutic strategies.

After the first studies by Dietrich et al. [113], it is now demonstrated that the anti-ferroptosis action of EU may be exploited against the Fe accumulation found to follow ischemic and hypoxic brain injuries [114] and in the management of ischemia/reperfusion injuries associated with ferroptosis [107,115]. The same strategy has been proposed to improve myocardial function by the EU-induced inhibition of the ROS-related ferroptosis signalling pathway [116].

With regard to the treatment of neurodegenerative diseases, a key step to counteract and possibly reverse neuro-behavioural abnormalities is to increase the presence of undeteriorated EU and to maintain it at a physiological level.

However, the replenishment of missing/damaged endogenous melanins with exogenous ones faces many difficulties, mainly due to the inability of the intravenously injected substances to cross the blood–brain barrier. Such obstacles are common to the whole melanin family, *in primis* to dopamine [107], and to overcome such issues, current research is being conducted to design a series of different formulations of melanin-derived nanomaterials [112,117].

**Figure 3:**
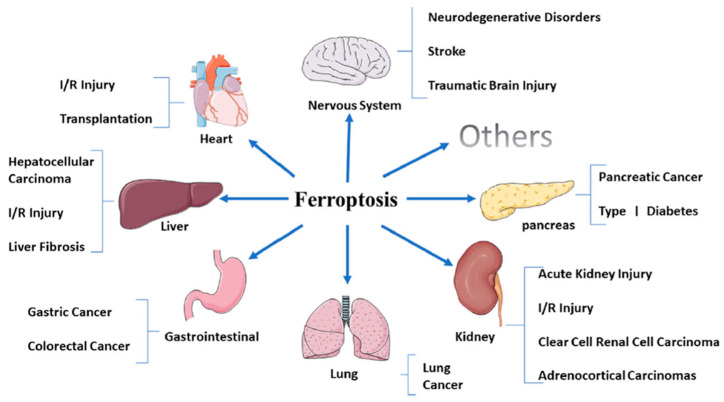
Organs and systems that are more vulnerable to ferroptosis-related diseases [Reproduced from Ref. [48] (open access)].

The rational engineering of EU-releasing systems is not limited to impart symptomatic relief to myocardial and neuronal dysfunctions induced by oxidative stresses because, as illustrated in Figure 3, several other organs and body systems are very vulnerable to ferroptosis [118,119]. 

In view of the ability to efficiently contrast any kind of oxidative stress, EU-based drugs and therapeutic agents are being increasingly applied in various biomedical areas [120].

Of particular interest is the application of EU and its derivatives in dermatology, where significant challenges include the regeneration of infected skin and problematic wounds, including burn wounds [121]. To improve the whole wound healing process, the request is for antibacterial, antioxidant, and conductive dressings able to promote angiogenesis and re-epithelialisation [122]. In this context, EU-based hydrogels have been demonstrated to meet all of the requirements for the treatment of severe skin injuries. The multi-tasking EU brings into play its multiple functionalities, from conductivity enabling cellular communication to an anti-inflammatory action enabling the normalisation of the secretion of inflammatory cytokines [123]. Moreover, EU-based hydrogels provide a moist environment, excellent haemostatic ability, enhanced cohesive and adhesive strength to biological tissues, and the feasibility of controlled drug delivery [124,125,126]. It is not surprising that a number of innovative EU-based surgical membranes have been introduced in clinical practice to treat all kind of burns, ulcers, and wounds.

Applications of EU-based materials are also proposed in dermo-cosmetics, where their photoprotective and antioxidant actions can help maintain healthy skin under a variety of harsh conditions [127]. A further, purely cosmetic application is the proposed substitution of the chemical dyes used to contrast the degradation of age-dependent hair pigmentation with EU-based formulations [128,129]. An additional advantage offered by the use of such bioinspired hair dyes is their proven antibacterial effects [130]. 

Nevertheless, one option that only this polymer can provide is the outstanding biological response to high-energy ionising radiation, a property that makes EU a key player in radiology and radiotherapy. The high-dose radiation utilised in some nuclear medical practices can alter the tissue microenvironment and affect both cell–cell interactions and intracellular signals [131]. In this context, a critical issue related to such irradiation treatments is the protection of the most radiosensitive tissues, such as hematopoietic tissue [132,133,134].

The internal administration of either natural or artificial EU-based formulations has been demonstrated to efficiently protect bone marrow hematopoietic cells during whole-body irradiation [135]. Due to this effect, it is thought possible to deliver higher radiation doses and, thus, to increase both the therapeutic and diagnostic efficacy of radiation-based medical treatments [15,136].

The internal use of EU as a novel radioprotective agent is also being investigated to contrast nuclear emergencies and to reduce the effects of cosmic rays in manned deep space flights and in the planned Moon and Mars colonisations [137].

However, further possibilities in therapeutic and diagnostic nuclear medicine are offered by the peculiar “attraction” of EU for the ionising radiation emitted from radionuclides. The use of autoradiographic techniques has, indeed, evidenced that injected radiolabelled compounds mostly accumulate in pigmented tissues [70]. This feature is being exploited in the preparation of radiopharmaceuticals and radiolabelled contrast compounds for targeted delivery and deposition into specific EU-enriched tissues.

Overall, EU’s ability to interact with ionising radiation provides different levels of radioprotection and can provide various benefits to living organisms, from radio-adaptation to the selective accumulation of radiolabelled drugs.

## 5. Synthesis Routes for New Applications

The addition/replacement of damaged endogenous EU with healthy exogeneous biomaterial provides a new way to prevent and treat several diseases. On the other hand, the challenges posed by this innovative healthcare approach means that the whole issue of EU production requires more thought. Novel medical practices demand functional molecules with well-defined chemical/physical features and, moreover, the feasibility to release such formulations where and when required.

In view of this, new paradigm efforts are been made to adjust the traditional techniques of EU extraction/purification from natural sources [138,139] or to develop innovative chemical synthesis techniques [140]. The conventional approaches are rather unsuitable for applications that need the use of a particular member of the “melanin” family, mainly because of the difficulties encountered in scaling up an appropriate synthesis process or in overcoming the issue of the low solubility of such biomaterials in both polar and apolar media [141].

A revolutionary way to obtain EU with features appropriate for personalised healthcare is provided by the nascent field of synthetic biology, which makes use of genetically engineered microorganisms, in primis bacteria, to selectively produce a series of functional biopolymers [142]. Bacterial polymers are biogenic materials suitable for a wide range of high-value applications, from biochemicals to cosmetics and biofuels [143].

Programmed bacterial biopolymers are finding extensive applications in biomedicine, where their coupling with structural materials is proposed to redesign the whole biomedical field, offering new hybrid materials for the next generation of diagnostics and therapeutics [144]. Recent outstanding examples include *E. coli* genetically programmed to secrete modified curli fibres able to interact with gastrointestinal tissues [145] or to release adhesive proteins able to promote cell attachment on hydrogel surfaces [146]. With regard to EU, it is well known that a variety of wild microbial taxa (*Streptomyces*, *Pseudomonas*, *Rhizobium*, *Bacillus*, *Trichoderma*, *Shewanella*, *Aspergillus*, *Aeromonas*, etc.) are able to secrete melanin biopolymers.

**Figure 4:**
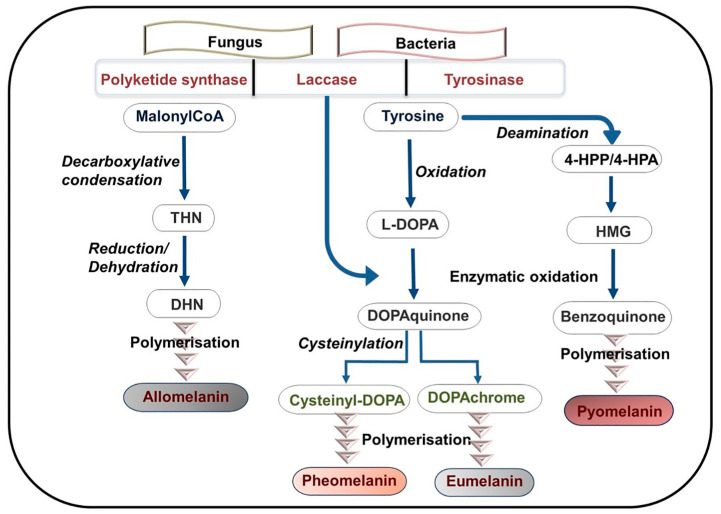
Scheme of general melanogenesis pathways in fungi and bacteria. Abbreviations: THN: 1,3,6,8-tetrahydroxynaphthalene, DHN: 1,8-dihydroxynaphthalene, L-DOPA: L-3,4-dihydroxyphenylalanine, HPP: hydroxyphenyl pyruvate, HPA: hydroxyphenyl acetate, HMG: 2,5-dihydroxyphenylacetate. (Reproduced with permission from Ref. [5]).

Figure 4 displays the melanogenesis pathways followed for the synthesis of the various melanins from fungi and bacteria. The biosynthesis of melanins from microorganisms starts from two main precursors, namely, tyrosine and malonyl CoA, with the latter one giving rise to DHN-type melanin through polyketide synthase. Conversely, the EU biopolymer is produced by synthesis routes that involve either laccase or tyrosinase. In particular, tyrosinase catalyses the oxidation of tyrosine and leads to the formation of extracellular eumelanin via the L-DOPA intermediate. 

A review of the potential offered by microbes in producing EU and other melanins for various therapeutical uses can be found in Ref. [5].

While the low yields of the pigment that can be obtained from wild bacteria make this production method unsuitable for most of the envisaged applications, the engineering of melanogenic bacteria strains provides a totally new scenario [147]. Microbial “cell factories” can allow the biosynthesis pathway to be controlled and the production of selected melanin types to be scaled [148].

In 1990, the first microbial melanin was obtained from a recombinant *E. coli* strain constructed by expressing a *Streptomyces antibioticus* gene [149]. Nowadays, the identification of viable genes and the characterisation of a large set of suitable bacteria enable the generation of an ever-increasing number of recombinant strains designed for the production of EU-based formulations with tailored functionalities [5].

This variety of available biological sources, on the one hand, allows the customisation of the type of microbial melanin that can be generated, and on the other hand, does not allow a standard production protocol to be defined. Depending on the choice of the recombinant microorganism, all of the culture parameters—media components, pH, temperature, aeration, light and radiation exposure—must be adapted to modulate the expression of genes and activate more efficient synthetic routes [150,151]. 

Increased melanin secretion can also be obtained from non-melanogenic microorganisms subjected to random mutagenesis. In one report, silent genes of melanin production, activated by a random mutation, allowed the mutant *Pseudomonas putida F6* strains to overproduce black pigment, consuming eight times less tyrosine with respect to the wild-type strain [152]. 

The increased biosynthesis yields, along with the feasibility to selectively obtain specific pigments by an appropriate choice of culture medium and fermentation conditions, are driving researchers to use bacterial EU in ever-wider biomedical fields [153].

Besides the already well-proven antimicrobial activity against both Gram-positive and Gram-negative pathogens [154], the antiproliferative properties make bacterial EU a promising anticancer agent. Examples are given by the EU obtained from a melanised *Bacillus licheniformis* strain as well as by EU generated from the *Streptomyces glaucescens* NEAE-H strain. The first one showed a therapeutical action comparable with that of doxorubicin against various cancer cell lines [155], and the second was demonstrated to contrast skin cancer cell lines better than conventional fluorouracil drugs [156].

Moreover, the unique capacity of bacterial EU to trigger biological reactions in response to environmental stimuli can be exploited to mitigate the dangerous effects produced by highly energetic ionising radiation. A recent paper reported that the administration of melanised *E. coli Nissle* to mice allows radiation-induced injuries of the gut mucosa to be prevented, helping to maintain intestinal homeostasis [157]. These findings highlight the role of bacterial EU in the development of mitigators able to contrast injuries and restore dysbiosis in cancer patients subjected to radiotherapies, in astronauts during long-term deep-space missions, and in populations exposed to high doses of ionising radiation in general. 

However, synthetic biology can also offer other advantages. As demonstrated by studies performed on genetically engineered *E. coli*, it is possible to programme microorganisms to not only produce EU with ad hoc tailored features, but also to secrete in situ, such as modified pigments in response to a specific stimulus [158]. The several interesting results obtained up to now indicate that the in situ programmable secretion of EU-based formulations is an achievable goal. This would make it possible to effectively exploit such multifunctional materials in personalised medicine and innovative diagnostics and the build-up of biosystems with autonomous biosensing/biodetection abilities [158].

## 6. Concluding Remarks

It is noteworthy that, for a long time, the chemical/structural complexity of diverse pigments known as “melanin” has prevented the large-scale production of such biopolymers with the chemical/physical features required for current biomedical applications, confining their use mainly to basic research.

This applies especially to EU, the most versatile and valuable member of the melanin family. Due to the variety of monomer ratio and building block stacking, dual ionic–electronic conductivity, and the complex system of free radicals and related multiple oxidation states, EU is, indeed, different from any other semiconducting polymer and is able to perform many different roles simultaneously. Although this makes endogenous EU a fundamental player in the physiology of the human body, any disturbance in melanogenesis results in the onset of more or less serious pathologies. The current hypothesis is that, besides skin disorders and dysfunctions of the visual and auditory systems, some significant neurodegenerative diseases—in primis AD, PD, HD, ALS, and FA—are also associated with EU abnormalities. 

In this context, the emerging approach to counter the effects provoked by qualitative or quantitative deficiencies of endogenous EU relies on the supplementation of artificially generated pigments with well-defined functionalities. 

A further challenge is the programmed delivery of such customised bio-inspired materials in those body parts that are in need. Today, this new administration method could be made possible by advances in biochemistry and the genetic engineering of bacteria and melanogenic microorganisms. 

Due to the large number of suitable or adaptable microorganisms, the bacterial synthetic routes provide chances to design, produce, and handle a variety of EU-based biomaterials with tailored features and are, therefore, able to respond to specific biological requirements. A further potential advantage of the EU obtained from genetically engineered organisms is the feasibility to be generated and released on-site.

Although the bio-technological route represents an alternative approach to extraction from natural sources or conventional chemical syntheses, not all of the problems associated with the biosynthetic production of EU have been solved so far. 

The feasibility of upscaling the pigment production is affected by the large number of parameters that play key roles in the fermentation process and by the consequent lack of a universal protocol for metabolic pathways. All of the EU-related issues highlighted in this review still present challenges that need to be addressed. As an example, to validate the feasibility of administering exogeneous EU to replace damaged or missing endogenous EU, the problem of the poor solubility of this polymer must be solved.

Taken as a whole, the enhanced production of EU-based formulations holds tremendous potential, either in therapeutics for the localised supplementation/modification of endogenous melanins, or in advanced diagnostics, with the perspective to combine multi-modal diagnostics and multi-therapeutic actions in a global approach. Decades of studies have allowed significant technological results to be obtained and the current developments in biotechnologies are allowing biogenic EU to cover increasingly important roles in green sciences/technologies and environmental recovery.

However, even if much has already been understood, there are still some important aspects of EU behaviour that need to be fully elucidated. EU is, indeed, characterised by an intriguing ability to reverse its functional processes, giving rise to unexpected adverse reactions. In this context, the stimulus–response mechanisms that occasionally trigger abrupt alterations of the EU redox equilibrium and switch the pigment behaviour from anti-oxidant to pro-oxidant still need to be understood. Examples are the surprising photoreactivity of the skin EU that is responsible for the onset of melanoma pathogenesis and the peculiar energy transfer from UVA-excited EU to DNA nucleotides.

Thus, the translation of EU in existing biosystems, regardless of how the pigment has been obtained, needs to take into account the fact that EU may put into action unexpected detrimental responses to some, so far unidentified, stimuli. 

The challenge is now to acquire the ability to predict the behaviour of EU under a variety of conditions and to manage the opening of its response pathways, selectively locking those that lead to adverse reactions and produce harmful effects.

## Figures and Tables

**Table 1:** Summary of the main diseases of the skin, eye, ear, and brain induced by the loss or degeneration of eumelanin.

Organ	EU Condition	Disease		Reference
**Skin**	Loss	Hypopigmentation	- Albinism	[59,60]
			- Vitiligo	[61,93]
	Proliferation	Hyperpigmentation		[94]
		Cutaneous melanomas		[17,58,95]
**Eye**	Partial Loss/Degeneration	Photophobia		[17,64,67]
		Visuospatial/visuoperceptual disturbances		
		Macular degeneration		[66,67]
		Cataract		
		Glaucoma		
		Retinopathies		
	Severe loss	Uveal melanoma		[96]
**Ear**	Partial Loss/Degeneration	Abnormal susceptibility to noise		[76,77]
		Reduced localization of sounds		
		Loss of equilibrium		
	Severe loss	Hearing loss		[17,67]
		Deafness		
**Brain**	Degeneration/Loss	Alzheimer’s disease		[82,83,84]
		Parkinson’s disease		[85,86,97,98]
		Huntington’s disease		[87]
		Amyotrophic lateral sclerosis		[88]
		Friedreich’s ataxia		[89]

## Data Availability

Not applicable.
